# Urinary Neutrophil Gelatinase-Associated Lipocalin (NGAL) and Cystatin C in Early Detection of Pediatric Acute Kidney Injury; a Diagnostic Accuracy Study

**Published:** 2018-01-12

**Authors:** Neamatollah Ataei, Sonbol Ameli, Mahmoud Yousefifard, Alireza Oraei, Fatemeh Ataei, Behnaz Bazargani, Arash Abbasi, Mostafa Hosseini

**Affiliations:** 1 Pediatric Chronic Kidney Disease Research Center, Tehran University of Medical Sciences, Tehran, Iran; 2 Department of Pediatric Nephrology, Children’s Hospital Medical Center, Tehran University of Medical Sciences, Tehran, Iran; 3 Physiology Research Center and Department of Physiology, Faculty of Medicine, Iran University of Medical Sciences, Tehran, Iran; 4 Department of Medicine, School of Medicine, Tehran University of Medical Sciences, Tehran, Iran; 5 Department of Nuclear Medicine, Department of Nuclear Medicine, Valiasr Hospital, Zanjan University of Medical Sciences, Zanjan, Iran; 6 Department of Epidemiology and Biostatistics, School of Public Health, Tehran University of Medical Sciences, Tehran, Iran

**Keywords:** Cystatin C, Lipocalin-2, Acute Kidney Injury, Early Diagnosis, Dimensional Measurement Accuracy

## Abstract

**Introduction:**

There is a controversy regarding accuracy of neutrophil gelatinase-associated lipocalin (NGAL) and Cystatin C in early detection of acute kidney injury (AKI). The present study aimed to compare the diagnostic value of two biomarkers in this regard.

**Method:**

In the present diagnostic accuracy study, all children between the ages of 1 month to 14 years were entered. Pediatric Risk, Injury Failure, Loss, End-stage renal disease (pRIFLE) criteria was used for identification of children with AKI as the reference test. Blood samples were taken from all patients at baseline and 48 hours after admission to assess serum creatinine and Cystatin C level. In addition, a urine sample was obtained within 6 hours of admission in order to measure NGAL level. In the end, area under the receiving operating characteristics (ROC) curve, sensitivity, and specificity of urine NGAL (uNGAL) and Cystatin C in early detection of AKI were compared.

**Results:**

Data from 96 children with the mean age of 27.31±36.24 months were entered (56.25% girls). Area under the ROC curve of uNGAL level in diagnosis of AKI in children was 0.91 (95% CI: 0.80 to 1.00) and area under the ROC of Cystatin C was 0.90 (95% CI: 0.77 to 1.00). Both tests had the same value in diagnosis of AKI (p=0.89). The best cut-off point of uNGAL for diagnosing AKI was 125 mg/L. uNGAL had a sensitivity and specificity of 0.92 (0.62 to 0.99) and 0.69 (0.57 to 0.78), respectively. The best cut-off point of serum Cystatin C level was 0.4 mg/L. Cystatin C had a sensitivity and specificity of 0.92 (0.62 to 0.99) and 0.64 (0.52 to 0.74), respectively.

**Conclusion:**

The present study showed that uNGAL level has the same value as serum Cystatin C level in early diagnosis of AKI.

## Introduction

Acute kidney injury (AKI) is considered an important issue that if not promptly managed can lead to serious complications such as chronic kidney disease, end-stage renal diseases and even death (1-3). Recent studies show that preventive strategies can significantly reduce the burden of the disease (4) and early diagnosis and treatment of AKI can significantly decrease medical costs. Unfortunately kidney diseases are asymptomatic most of the time and disease manifestations do not occur until a large amount of renal tissue is destroyed (5). Hence, the best suggestion is that nephrologists and physicians of other specialties begin to comprehend the importance of early diagnosis of kidney disease.

Several diagnostic methods are introduced for identification of children with AKI, but none of them could accurately predict the outcome of disease in the initial stages (6). Over the past decade, many studies have shown that multiple serum and urine biomarkers are useful for early detection of kidney injury and have a better prognostic role than previous diagnostic tools (7-9). After three meta-analyses we reached a conclusion indicating that biomarkers such as neutrophil gelatinase-associated lipocalin (NGAL) and Cystatin C are accurate predictors of AKI (10-12). However, it is yet to be studied which one of these biomarkers and their sources (serum or urine) have greater value in prediction of AKI. A meta-analysis reported that serum Cystatin C has a greater diagnostic value in detection of AKI in children and adolescents than its urine level (10). Existing evidence regarding NGAL is however inconclusive and further research needs to be carried out (13-15). The present study aimed to assess the value of urinary NGAL (uNGAL) and serum Cystatin C in early diagnosis of hospitalized children with AKI.

## Methods

***Study design and setting***

In the present diagnostic accuracy study, the value of urine NGAL in early detection of pediatric AKI was assessed. All children brought to Children’s Medical Center of Tehran, Iran, between 2015 and 2016 were entered in a prospective manner. The protocol of the present study was approved by the Ethics Committee of Tehran University of Medical Sciences and all researchers adhered to the principles of the Helsinki declaration during the study period. Additionally, a consent form was obtained from patients or their parents.

***Participants***

The study population of the present study consisted of all children between the ages of 1 month and 14 years, who were suspected to AKI according to their in-charge physician’s opinion. Exclusion criteria were children with chronic kidney disease, need for renal replacement therapy and those not giving consent. Convenience sampling was used in the present study.

***Test methods***

Blood samples were taken from all patients on admission and 48 hours after admission and were sent to the laboratory of Children’s Medical Center in order to assess serum creatinine and Cystatin C level. Creatinine measurement was done using Jaffe method, a spectrophotometry kit and a Hitachi 717 device. Additionally, serum Cystatin C was measured using Cystatin C ELISA Kit from BioPorto Diagnostic of Denmark using Sandwich ELISA method. Glomerular filtration rate (GFR) was calculated based on Cystatin C formula (16).

A urine sample was obtained within 6 hours of admission and was stored in -80°c after centrifuge. Measurement of uNGAL was done using an NGAL ELISA kit from BioPorto Diagnostics, Denmark using Sandwich ELISA method. In addition, the laboratory and technicians who entered data were not aware of the AKI status of patients. Index test was assessed in a blind manner as uNGAL measurement was performed before GFR calculation.

***Outcome***

AKI was considered as the endpoint of the study. Pediatric Risk, Injury Failure, Loss, End-stage renal disease (pRIFLE) criteria was used for identification of children with AKI as the reference test (17). Therefore, a decrease in estimated creatinine clearance by at least 25% from baseline within 48 hours after admission was considered the definition of AKI.

***Statistical analysis***

The required sample size was estimated based on the suggested method of Hajian-Tilaki (18). A sensitivity of 93% for uNGAL in diagnosis of AKI and a minimum marginal error of 0.1 (19) were considered in calculating the sample size hence, 91 patients were required. In the end, data from 96 patients were entered and analyzed.

Data were analyzed using STATA 11.0. Patients were divided to two groups of AKI and no-AKI based on their GFR level within 48 hours of admission. At the end, area under the receiving operating characteristics (ROC) curve, sensitivity, specificity, positive predictive value (PPV), negative predictive value (NPV), positive and negative likelihood ratios of uNGAL and Cystatin C were calculated in order to assess their discriminatory power with a 95% confidence interval (CI). Areas under the curve of the two biomarkers were compared using the method suggested by Cleves and Rock (20). 

General calibration was assessed by drawing calibration plot, which showed that the perfect calibration was the reference line with an intercept of zero and slope of 1. As the slope and intercept of calibration plot of models become closer to 1 and zero, respectively, they will be considered more perfect in prediction of AKI. Additionally, overall performance was calculated using Brier score in order to assess the predictive accuracy and predictive reliability. A p value of less than 0.05 was considered significant in all analyses.

## Results

***Baseline characteristics***

Data from 96 children with the average age of 27.31±36.24 months (ranging from 1 month to 156 months) were entered (56.25% boys). Urologic problems (26.96%) were the most common etiology for AKI. Due to pRIFLE criteria, 13 cases (13.54%) of AKI were identified. The uNGAL level in AKI group (1929.92±3957.54) was significantly higher than no-AKI group (168.59±284.52) with a p value of less than 0.0001. Similar results were observed regarding Cystatin C levels (1.97±1.10 vs. 0.35±0.45; p < 0.0001) (table 1).

**Figure 1:**
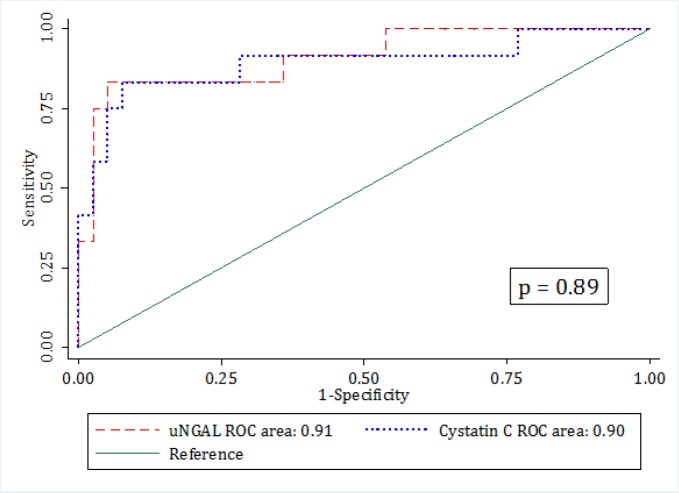
Comparison of the areas under the receiver operating characteristics (ROC) curve of urinary neutrophil gelatinase associated lipocalin (uNGAL) and serum Cystatin C level in diagnosis of acute kidney injury in children within 48 hours of admission

**Figure 2:**
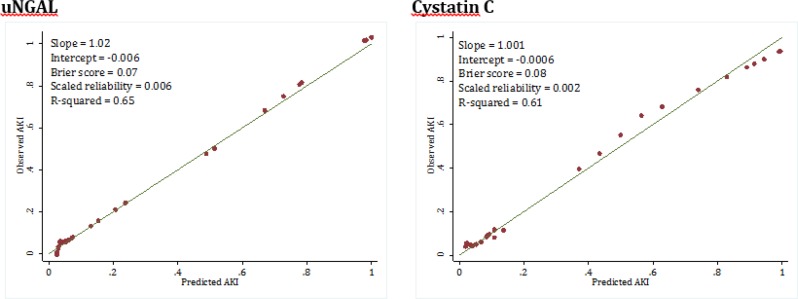
Calibration plot of urinary neutrophil gelatinase-associated lipocalin (uNGAL) and Cystatin C in detection of acute kidney injury (AKI).

Area under the ROC of urine NGAL level in diagnosis of AKI in children was 0.91 (95% CI: 0.80 to 1.00) and area under the ROC of Cystatin C was 0.90 (95% CI: 0.77 to 1.00). Both tests had the same value in diagnosis of AKI (p=0.89) (figure 1). Both biomarkers had appropriate calibration levels. The slope of calibration line for uNGAL and Cystatin C were 1.02 and 1.001, respectively. The calculated intercept of the mentioned biomarkers were -0.006 and -0.0006, respectively. Additionally, Brier score of uNGAL and Cystatin C were 0.07 and 0.08, respectively, which showed good overall performance of both biomarkers in diagnosis of AKI in children (figure 2).

The best cut-off point of uNGAL for diagnosis AKI was 125 mg/L. uNGAL had a sensitivity and specificity of 0.92 and 0.69, respectively at this level. uNGAL level had a PPV and NPV of 0.32 and 0.98, respectively. The best cut-off point of serum Cystatin C level was 0.4 mg/L. Cystatin C had a sensitivity and specificity of 0.92 and 0.64, respectively at the mentioned cut-off point. Additionally, Cystatin C had a PPV and NPV of 0.28 and 0.98, respectively (table 2).

**Table 1:** Summary of demographic, baseline, and laboratory values based on outcome

**Variable**	**No-AKI** **(n = 83)**	**AKI** **(n = 13)**	**Total** **(n = 96)**	**P**
**Sex (n, %)**				
Boy	47 (56.63)	7 (53.85)	54 (56.25)	0.85
Girl	36 (43.37)	6 (46.15)	42 (43.75)	
**Age (month)**	28.66±37.58	18.69±25.58	27.31±36.24	0.64*
**Etiology of AKI**				
Pneumonia	7 (8.43)	0 (0.0)	7 (7.29)	0.24^#^
Cardiac diseases	14 (16.87)	3 (23.08)	17 (17.71)	
Trauma	3 (3.61)	0 (0.0)	3 (3.13)	
Sepsis	4 (4.82)	2 (15.38)	6 (6.25)	
Urologic disorders	21 (25.30)	2 (15.38)	23 (23.96)	
Pulmonary	14 (16.87)	2 (15.38)	16 (16.67)	
Liver diseases	6 (7.23)	2 (15.38)	8 (8.33)	
Metabolic disorders	3 (3.61)	2 (15.38)	5 (5.21)	
Other	11 (13.25)	0 (0.0)	11 (11.46)	
**Height (cm)**	76.48±25.30	72.08±21.55	75.88±24.75	0.63 *
**Cystatin C on admission (mg/L)**	0.35±0.45	1.97±1.10	0.57±0.80	< 0.0001*
**uNGAL (mg/L)**	168.59±284.52	1929.92±3957.54	407.10±1554.12	< 0.0001 *
**Creatinine (mg/L) **				
Baseline	0.48±0.16	0.49±0.16	0.49±0.16	0.61 *
48 hours	0.44±0.14	1.08±0.36	0.53±0.28	< 0.0001 *
**eCCI (ml/min/ 1.73 m2)**				
Baseline	91.36±37.24	34.46±10.84	83.66±39.94	0.61 *
48 hours	91.36±37.24	34.46±10.84	83.66±39.94	< 0.0001 *

AKI: acute kidney injury; eCCI: estimated creatinine clearance by Cystatin C equation; uNGAL: urinary neutrophil gelatinase-associated lipocalin.

* , based on Mann-Whitney U test;

# , based on Fisher’s exact test.

**Table 2:** Screening performance characteristics of urinary neutrophil gelatinase-associated lipocalin (uNGAL) and Cystatin C in detection of acute kidney injury (AKI)

Performance	Cystatin C	uNGAL
Cut off	0.4 mg/L	125 mg/L
True positive	12	12
True negative	53	57
False positive	30	26
False negative	1	1
Sensitivity	0.92 (0.62 to 0.99)	0.92 (0.62 to 0.99)
Specificity	0.64 (0.52 to 0.74)	0.69 (0.57 to 0.78)
Positive predictive value	0.29 (0.16 to 0.45)	0.32 (0.18 to 0.49)
Negative predictive value	0.98 (0.89 to 1.0)	0.98 (0.89 to 1.0)
Positive likelihood ratio	2.55 (1.84 to 3.54)	2.95 (2.07 to 4.20)
Negative likelihood ratio	0.12 (0.02 to 0.80)	0.11 (0.02 to 0.74)

* , Data are presented as estimated value and 95% confidence interval.

## Discussion

The present study showed that uNGAL and serum Cystatin C level are reliable biomarkers for early detection of pediatric AKI. Level of these biomarkers measured early after injury can identify AKI much sooner than conventional methods.

NGAL is a 20 kDa protein expressed in renal tubules which is released into the urine in case of acute or chronic kidney injury. Therefore, uNGAL level might reflect kidney injury. Faster increase in uNGAL level compared to serum creatinine level in response to AKI is one of its benefits over creatinine. However, some biomarkers such as serum creatinine level indicate renal function and are indirectly indicative of AKI (21). In other words, GFR should decrease to a certain level in order to disturb creatinine clearance and increase its serum level, which is required for diagnosis of AKI. However, NGAL level increases in urine in response to kidney injury; hence, it can be a better marker of the severity of AKI (22). In a meta-analysis of 37 studies by researchers of the present study, it was reported that assessing uNGAL level within the first 6 hours of admission can accurately predict the incidence of acute kidney injury in children in the first 48 hours (12). Similar results were found in another meta-analysis on serum NGAL level. However, the best window period for measuring serum NGAL level was reported to be within the first 12 hours of admission (11). Due to the mentioned findings, uNGAL level seems to have a better performance in prediction of AKI. 

Cystatin C is another biomarker, the predictive value of which was proven in the present study. Similarly, a meta-analysis showed that serum Cystatin C level has a reasonably good value in predicting AKI in children. Additionally, serum Cystatin C level has a greater value compared to its urine level (10). Hence, serum Cystatin C level was used in the present study. For the first time, the present study showed that uNGAL and serum Cystatin C level have similar values in prediction of AKI in children.

***Limitations***

Small sample size is one of the limitations of the present study. In the present small sized study, patients brought to an individual center were assessed and selection bias might be present as convenience sampling was used. Short follow-up period (48 hours) was another limitation of this study. Additionally, misclassifications might be present as a small number of patients show signs and symptoms of AKI (based on pRIFLE criteria) after the first 48 hours (during the first week of admission or even later).

## Conclusion

The present study showed that both uNGAL and serum Cystatin C level are highly valuable predictors of AKI within the first hours of admission. In other words, level of these biomarkers measured early after injury can identify AKI much sooner than conventional methods. Additionally, uNGAL seems to be a better biomarker in children and adolescents as its assessment is less invasive than assessing serum Cystatin C level and can be measured using a single urine sample.
